# CHronic hypERtension and L-citRulline studY (CHERRY): an Early-Phase Randomised Controlled Trial in Pregnancy

**DOI:** 10.1007/s43032-023-01335-4

**Published:** 2023-10-03

**Authors:** Laura Ormesher, Stephanie A. Worton, Ashley Best, Susanna R. Dodd, Alice Dempsey, Elizabeth C. Cottrell, Heather Glossop, Catherine Chmiel, Hoi Yee Wu, Ben Hardwick, Sophie Hennessy, Edward D. Johnstone, Jenny E. Myers

**Affiliations:** 1 Manchester University Hospitals NHS Foundation Trust, Manchester, UK; 2 Maternal and Fetal Health Research Centre, Division of Developmental Biology and Medicine, University of Manchester, Manchester, UK; 3 Liverpool Clinical Trials Centre, University of Liverpool, Liverpool, UK

**Keywords:** Hypertension, Pregnancy, Citrulline, Asymmetric dimethylarginine

## Abstract

Oral supplementation with L-citrulline, which is sequentially converted to L-arginine then nitric oxide, improves vascular biomarkers and reduces blood pressure in non-pregnant, hypertensive human cohorts and pregnant mice with a pre-eclampsia-like syndrome. This early-phase randomised feasibility trial assessed the acceptability of L-citrulline supplementation to pregnant women with chronic hypertension and its effects on maternal BP and other vascular outcomes. Pregnant women with chronic hypertension were randomised at 12–16 weeks to receive 3-g L-citrulline twice daily (*n* = 24) or placebo (*n* = 12) for 8 weeks. Pregnant women reported high acceptability of oral L-citrulline. Treatment increased maternal plasma levels of citrulline, arginine and the arginine:asymmetric dimethylarginine ratio, particularly in women reporting good compliance. L-citrulline had no effect on diastolic BP (L-citrulline: − 1.82 *95% CI* (− 5.86, 2.22) vs placebo: − 5.00 *95% CI* (− 12.76, 2.76)), uterine artery Doppler or angiogenic biomarkers. Although there was no effect on BP, retrospectively, this study was underpowered to detect BP changes < 9 mmHg, limiting the conclusions about biological effects. The increase in arginine:asymmetric dimethylarginine ratio was less than in non-pregnant populations, which likely reflects altered pharmacokinetics of pregnancy, and further pharmacokinetic assessment of L-citrulline in pregnancy is advised.

**Trial Registration **EudraCT 2015-005792-25 (2017-12-22) and ISRCTN12695929 (2018-09-20)

**Graphical Abstract:**

In pregnant women with chronic hypertension, L-citrulline is an acceptable intervention which increased plasma L-citrulline bioavailability but did not affect BP, potentially due to altered pharmacokinetics of pregnancy.

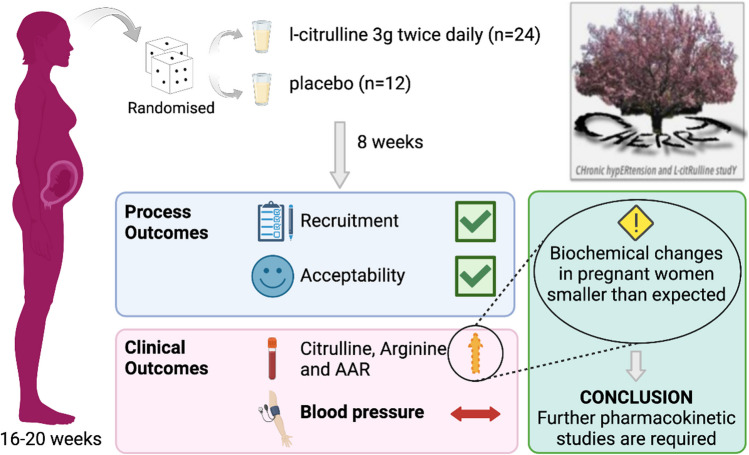

**Supplementary Information:**

The online version contains supplementary material available at 10.1007/s43032-023-01335-4.

## Introduction

Hypertensive disorders of pregnancy (HDPs), comprising chronic hypertension, gestational hypertension and pre-eclampsia/eclampsia, affect around 1 in 10 pregnancies [1]. HDPs carry significant risks for both mother and baby, increasing the risk of premature delivery and caesarean section rates in the mother and increasing perinatal mortality and foetal growth restriction for the foetus [2, 3].

Abnormal endothelial function and impaired nitric oxide (NO) pathways are strongly implicated in the pathology of HDPs [4, 5]. Endothelium-derived NO, which is produced from L-arginine by endothelial NO synthase (eNOS), is a key regulator of vascular tone and placental angiogenesis. Reduced NO bioavailability is associated with elevated blood pressure (BP) in human and animal studies both during and outside of pregnancy [6–9]. Asymmetric dimethylarginine (ADMA) is an endogenous inhibitor of eNOS activity and competes with L-arginine for endothelial cell uptake, both of which decrease NO synthesis [10, 11]. ADMA concentration is increased in an array of cardiovascular disorders, including in women with hypertension [12], obesity [13] and pre-eclampsia [14, 15]. The arginine:ADMA ratio (AAR) has a greater influence on NO production than arginine levels alone [16]. Supplementation with the eNOS substrate L-arginine increases the AAR and thereby increases NO availability improving vascular function [17]. A systematic review [18] suggested benefits of L-arginine supplementation in pregnant women with HDP but included women with both chronic and gestational hypertension. Furthermore some studies used L-arginine supplementation later in pregnancy, potentially after the best opportunity to prevent NO dysfunction in pre-eclampsia [5].

L-arginine is subject to extensive elimination by gut bacteria and systemic arginases and in the case of pregnancy by placental arginases. Oral L-citrulline is less subject to degradation as it bypasses gastrointestinal and liver metabolism [19] but is subsequently converted to L-arginine in the kidneys and vascular endothelium [20, 21]. Administration of oral L-citrulline is more effective at increasing plasma L-arginine than an equivalent dose of L-arginine in healthy subjects [22]. L-citrulline supplementation is associated with an increased arginine/ADMA ratio and improved vascular assessments [22, 23]. In a pre-eclampsia-like mouse model, L-citrulline supplementation throughout pregnancy improved maternal BP and vascular function [24]. L-citrulline has previously been used as an intervention in human pregnancy to increase AAR (NCT00743210) [25]. In this phase I study of 24 obese pregnant women, 3 weeks’ treatment with L-citrulline (3 g/day) was associated with a significant reduction in BP, which persisted after treatment cessation until the end of pregnancy [25].

Amino acid supplements, which are readily available and relatively low-cost, present an appealing intervention for HDP, where treatment options are limited. To our knowledge at present, there is no information regarding either the efficacy or acceptability of L-citrulline supplementation to modulate cardiovascular function in women with chronic hypertension in early pregnancy.

The aims of this early phase feasibility randomised trial were therefore (i) to assess the acceptability of L-citrulline supplementation during pregnancy in women with chronic hypertension, (ii) to determine the effect of L-citrulline supplementation on maternal BP and (iii) to determine whether L-citrulline supplementation is associated with a change in uterine blood flow and other markers associated with adverse pregnancy outcomes.

## Methods

Ethical approval was obtained from North West Haydock Research Committee (16/NW/0557). The trial protocol was approved a priori by the Medicines and Healthcare products Regulatory Agency, the Trial Management Team, Trial Steering Committee and Data Monitoring Committee, and the trial was registered prospectively (EudraCT 2015-005792-25 and ISRCTN12695929). Trial management was overseen by the Liverpool Clinical Trials Research Centre, University of Liverpool. Adverse events were assessed by the study team and Trial Sponsor as described in the trial protocol.

Women referred to the Manchester Antenatal Vascular Service (MAViS) translational research clinic with a diagnosis of chronic hypertension were recruited following informed consent. Inclusion criteria were as follows: viable singleton pregnancy; gestation 12^+0^–16^+0^ weeks; age ≥ 18 years; diastolic BP ≥ 90 mmHg (average of two clinic readings) OR ≥ 80 mmHg if taking antihypertensive medication OR pulse wave velocity (PWV) ≥ 9 m/s before 16 weeks’ gestation; and serum creatinine < 120 mmol/l at booking. Within our local population, women meeting these criteria have a 43% risk of developing a pregnancy complication necessitating early delivery (*OR* 2.9, *95% CI* (1.45, 5.83); unpublished data J Myers). A double-blinded design was implemented with concealment of allocation until completion of pre-specified statistical analysis; a block randomisation list was held by the Trial Pharmacy and following consent, women were randomised to receive 30 ml L-citrulline solution (10% solution containing 3 g L-citrulline; Stockport Pharmaceuticals, Stockport, UK) twice daily or matched placebo (Stockport Pharmaceuticals) from randomisation to 22 ± 2 weeks (maximum duration 10 weeks). Allocation was 2:1 active vs placebo to maximise efficacy data collection.

At baseline (pre-treatment; Visit 1), and at 4 weekly study visits (Visit 2 during treatment and Visit 3 at the end of the treatment period), side effects were reviewed, blood samples were obtained, and clinical measurements were recorded: BP (× 3 at rest; Alere Microlife, Waltham, USA), PWV via cuff-based oscillometry arteriograph (TensioMed Ltd., Budapest, Hungary), cardiac output and peripheral vascular resistance (Non-Invasive Cardiac Output Monitor (NICOM); Cheetah Medical, Watford, UK) and uterine artery Doppler. Ambulatory BP monitoring (ABPM) was fitted for 24 h at Visits 1 and 3. To assess compliance at Visits 2 and 3, women were asked to report how often they had missed treatment doses and to return bottles for estimation of discrepancies between the volume of study drug utilised and the reported number of missed doses. The percentage of missed doses was estimated as (100 × number of reported missed doses) / expected number of doses between baseline and the week 8 visit, to account for participants whose Visit 3 was later or earlier than the specified 8 weeks. Due to the 2-week window for visits, some women completed their allocated treatment several days prior to Visit 3. At the end of the treatment period, women completed an acceptability questionnaire. All study measurements were performed at St Mary’s Hospital, Manchester. All measurements, participant characteristics and pregnancy outcomes were recorded using a secure Web-based database (COLLECT; MedSciNet) [26].

Blood samples collected in serum gel and EDTA tubes were stored on ice and centrifuged within 4 h at 4 °C for 10 min at 3000 rpm, prior to aliquoting and storage at − 80 °C for future biochemical analysis. Plasma samples were deproteinised using sulphosalicylic acid solution containing norvaline as internal standard, and the free amino acids were derivatised using AccQ-Tag Ultra reagent (6-aminoquinolyl-N-hydroxysuccinimidyl carbamate; Waters Corporation, Milford, USA). The derivatives were separated with gradient elution on an AccQ-Tag Ultra column (Waters Corporation) using the Waters Acquity UPLC system with UV detection at 260 nm. Batched enzyme-linked immunosorbent assays were performed on serum samples at the end of the study according to manufacturer instructions to quantify placental growth factor (PlGF; R&D Systems, Minneapolis, USA), soluble fms-like tyrosine kinase 1 (sFlt-1; R&D Systems) and ADMA (Immundiagnostik AG, Bensheim, Germany).

### Study Outcomes

The data that support the findings of this study are available from the corresponding author upon reasonable request. All outcomes were pre-specified in the trial protocol. The primary process outcome was recruitment rate (number of women eligible, recruited and completing study per month). The primary clinical outcome was reduction in diastolic BP from baseline to 8 weeks post commencement of L-citrulline supplementation.

The secondary process outcome was acceptability of the intervention in pregnant women with chronic hypertension. Secondary clinical outcomes were as follows: (i) change in maternal haemodynamic measurements (PWV, augmentation index, central BP and total peripheral vascular resistance) following L-citrulline supplementation; (ii) change in uteroplacental blood flow indices; (iii) change in maternal plasma AAR and angiogenic markers.

### Sample Size

The target sample size was calculated using existing BP data from the ongoing MAViS cohort study (11/NW/0426; *n* = 230), which indicate the mean change in diastolic BP between 14–16 weeks’ and 22–24 weeks’ gestation is − 0.1 ± 9.8 mmHg. This feasibility study was powered to detect a reduction in clinic diastolic BP of 6 mmHg in line with the findings of previous studies in pregnancy using L-citrulline supplementation ([25]; change in mean arterial pressure 8 ± 6 mmHg, *n* = 24). A sample size of 24 women in the active treatment (L-citrulline) group would be sufficient (with 80% power and a 95% level of confidence) to detect a 6 mmHg change (within group), assuming a SD of 10 mmHg. A recruitment target of 42 women was set to allow for 10% non-completion. Analysis for secondary endpoints was pre-specified but not powered. An exploratory analysis to investigate the association between plasma AAR with BP, PWV and uterine artery Doppler was also included in the study protocol.

### Statistical Analysis

A statistical analysis plan was agreed by the Trial Management Team and Trial Statistical team prior to analysis. The principle of intention-to-treat was adopted for primary and secondary outcomes, and no interim analysis was planned. Analyses included all randomised participants for whom the outcome(s) of interest (and any covariates) were available. Categorical data are presented using counts and percentages. Continuous data are presented as mean (standard deviation) and median (Quartile 1, Quartile 3) for normally and non-normally distributed variables, respectively. Primary and secondary clinical outcomes were calculated as the within group difference in measurements obtained at the baseline visit (Visit 1) and end of treatment (Visit 3) and presented as the absolute difference and standardised difference (divided by the number of days between visits). An exploratory analysis investigated the between group difference in all cardiovascular outcomes between baseline (Visit 1) and end of treatment (Visit 3) using standard analysis of covariance with the baseline measurement, treatment allocation and days between visits included as covariates.

A number of post hoc analyses are included here which were not included in the original statistical analysis plan. These include systolic BP, pregnancy outcomes and longitudinal measurement of BP, uterine artery and angiogenic markers after treatment cessation in routine clinical visits in the translational research clinic. In addition, analysis of the arginine, L-citrulline and ADMA measurements and the relationship of these measurements to cardiovascular parameters and compliance were a post hoc analysis. All post hoc analyses were performed by the research team (JEM), not the Liverpool Clinical Trial Centre statistician. Reporting of the incidence of pre-existing diabetes, parity and baseline antihypertensive use within the study population was not pre-specified.

## Results

### Process Outcomes

**Fig. 1:**
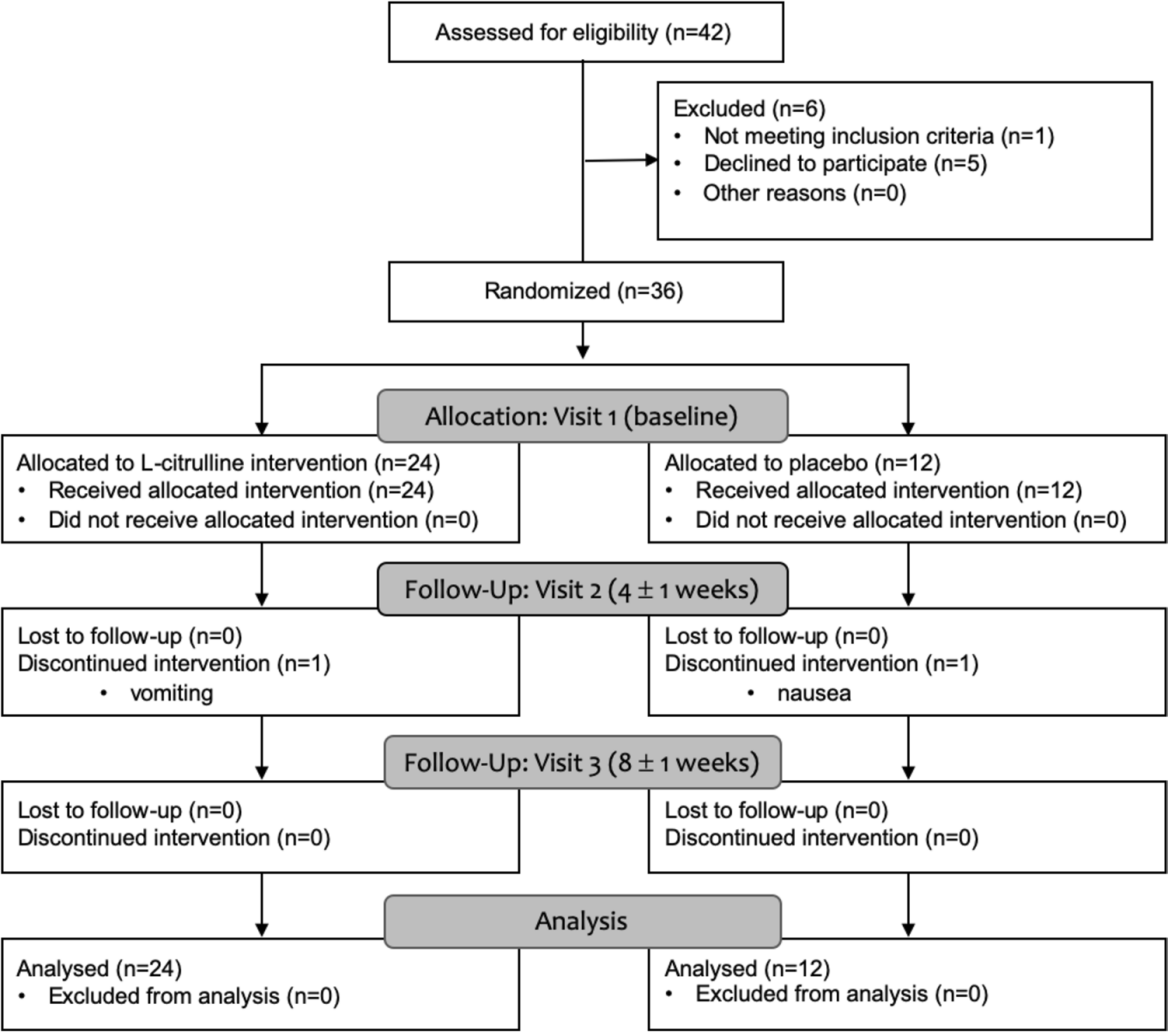
Consort flow diagram

Between July 2017 and January 2018, 42 women were screened and 36 women were recruited and randomised (5.1 women/month); the recruitment rate was 87.8%. Despite target recruitment of 42 women, recruitment had to be capped at 36 women (the target completed sample size) due to issues related to L-citrulline/placebo stock availability and storage restrictions. This was partially mitigated by a lower than anticipated drop-out rate. The consort diagram is shown in Fig. 1 and characteristics of the study population are shown in Table 1. Two women (one placebo, one L-citrulline) discontinued treatment 4 weeks into the study due to reasons including vomiting, nausea and dislike of the taste; these women continued to attend study visits, and their data were included in analysis. Four adverse events were recorded (Online Resource 1), including one serious adverse event (foetal ventriculomegaly at 33 weeks), which was not thought to be related to study participation.

**Table 1:** Characteristics of study population

Variable		L-Citrulline (*n* = 24)	Placebo (*n* = 12)
Age (years)		33.8 [31.1, 35.6]	36.0 [32.7, 37.7]
BMI (kg/m^2^)		29.0 [28.0, 33.0]	31.5 [25.3, 42.2]
Systolic BP (mmHg)		130.7 [122.5, 135.8]	138.7 [124.5, 152.5]
Diastolic BP (mmHg)		85.8 [81.7, 93.0]	93.0 [86.7, 99.2]
Years since diagnosis (years)		3.4 [1.0, 10.2]	4.7 [1.46, 9.7]
Gestational age at recruitment (days)		91.0 [87.5, 98.5]	95 [93.5, 99.5]
Ethnicity	Black African	6 (25.0%)	2 (16.7%)
	Black Caribbean	1 (4.2%)	2 (16.7%)
	East/Central Asian	2 (8.3%)	0 (0.0%)
	Others	1 (4.2%)	0 (0.0%)
	South Asian	2 (8.3%)	0 (0.0%)
	White	12 (50.0%)	8 (66.7%)
Hypertension diagnosis	Primary	19 (79.2%)	11 (91.7%)
	Secondary	4 (16.7%)	1 (8.3%)
Antihypertensive treatment last 12 m		15 (62.5%)	6 (50.0%)
Antihypertensive therapy at baseline	None CCB Alpha/beta blocker Combination	8 (33.3%) 10 (42.7%) 5 (20.8%) 1 (4.2%)	6 (50.0%) 3 (25.0%) 1 (8.3%) 2 (16.7%)
Nulliparous		6 (25.0%)	0 (0.0%)
Pre-gestational diabetes		3 (12.5%)	3 (25.0%)
Presence of proteinuria		2 (8.3%)	0 (0.0%)

Median [Q1, Q3] or number (%). CCB calcium channel blocker (nifedipine or amlodipine); alpha/beta blocker = labetalol

#### Assessments of Acceptability and Compliance

Acceptability and compliance were assessed at the end of the 8-week treatment period (Online Resource 2). Most women (26/32; 81.3%) found the intervention “easy”, whilst only 2/32 women (6.3%) found treatment “difficult”. On verbal recall, 11/32 women (34.4%) reported missing doses “once/twice per week” or “every day” (Table S2). Overall, the median [IQR] percentage of missed doses was 4.8 (1.9–14.3%) and was not different between active and placebo groups (Online Resource 3). The return of treatment bottles was insufficiently consistent to accurately assess the remaining volume of drug/placebo.

**Fig. 2:**
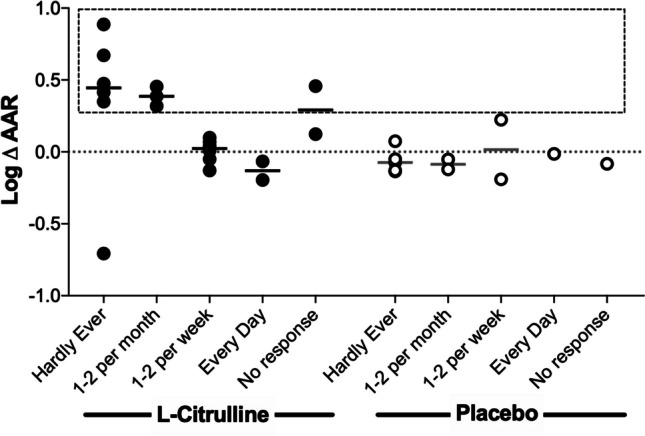
Change in arginine:ADMA ratio (AAR) in participants allocated to L-citrulline or placebo according to self-reported compliance. Log ΔAAR of women receiving L-citrulline or placebo. Values displayed according to the self-reported frequency with which doses were missed. The dashed box highlights L-citrulline-treated participants (9/20 with measurements) who had an increase in ΔAAR above that measured in any women allocated to the placebo treatment

The biochemical measurements of citrulline, arginine, ADMA and the calculated AAR at baseline, 4 weeks (Visit 2) and at the end of treatment (Visit 3) are shown in Online Resource 4a-d. Citrulline and arginine were both significantly increased in L-citrulline-treated women compared to placebo. There was a statistically significant correlation between arginine and citrulline concentrations at Visit 2 (0.37 [*95% CI* 0.23–0.52]; *R*^2^ = 0.45, *p* < 0.001) and Visit 3 (0.51 [0.38–0.63]; *R*^2^ = 0.67, *p* < 0.001) which was not present at baseline (0.44 [− 0.22–1.09]). ADMA concentrations were not significantly associated with either arginine or citrulline concentrations at any visit, and ADMA levels were not different between treatment groups (0.44 [0.42–0.47] vs 0.41 [0.37–0.45]; *p* = 0.1). At Visit 2 (during treatment), median AAR was increased in the treatment group (Table 2). This difference was not present at Visit 3 (Table 2). Only 6/20 women allocated to L-citrulline treatment with samples available achieved an AAR increase of 50% at 4 weeks (Online Resource 4). However, when considering the effect of compliance, women allocated to L-citrulline who reported good compliance (missed 1–2 doses per month or less) had a significantly higher (log) delta AAR measured during the treatment period (L-citrulline 9/24 women, median [min, max] 0.41 [− 0.71, 0.89] vs placebo 7/12 women − 0.074 [− 0.16, 0.075]; *p* = 0.008; Fig. 2).

**Table 2:** Comparison of arginine:ADMA ratio (AAR) between treatment groups

AAR	L-Citrulline			Placebo			*p*
	*Median*	*IQR*	*N*	*Median*	*IQR*	*N*	
Baseline	135.61	113.5, 173.1	20	138.3	133.7, 150.8	11	0.80
Visit 2	158.84	131.0, 192.0	23	129.8	108.8, 147.5	12	**0.03**
Visit 3	118.79	99.3, 181.3	21	124.4	109.3, 154.6	9	0.98
Visit 2 fold change	1.12	1.0, 1.5	20	0.9	0.9, 1.0	11	**0.01**
Visit 3 fold change	1.04	0.8, 1.4	17	0.9	0.8, 1.1	8	0.48

*p*<0.05 considered statistically significant

AAR at each visit and within-group fold change between visits compared between L-citrulline- and placebo-treated groups

### Clinical Outcomes

Pregnancy outcomes are summarised in Online Resource 5. Pre-eclampsia incidence was 22% (8/36 women; L-citrulline 5/24 (21%), placebo 3/12 (25%)). Birth weight centiles and delivery gestations were comparable between groups (not formally tested). Changes to antihypertensive medication during the study period are shown in Online Resource 6.

**Fig. 3:**
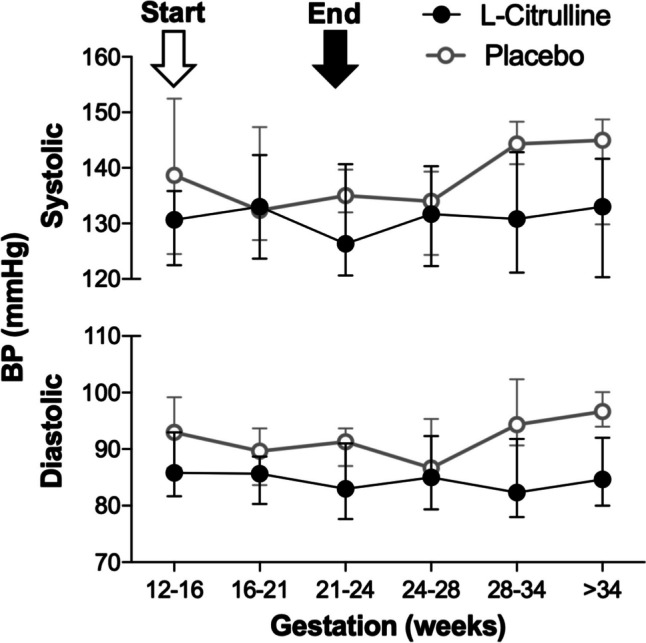
Systolic and diastolic BP across gestation in women allocated to L-citrulline (*n* = 24) and placebo (*n* = 12). Data shown as median [IQR]. Arrows indicate the start of treatment after the baseline visit and the end of treatment at/before Visit 3. Measurements taken beyond 24 weeks were recorded during routine clinical care visits

There was no change in diastolic BP from baseline to Visit 3 (L-citrulline − 1.82 [*95% CI* − 5.86, 2.22], placebo − 5.00 [− 12.76, 2.76] and L-citrulline − 0.03 [− 0.1, 0.03], placebo − 0.08 [− 0.21,0.05]; expressed as the absolute and standardised difference, respectively). Absolute and standardised systolic BP were also similar (1.17 [− 4.64, 6.97] vs − 2.28 [− 11.21, 6.51] and 0.13 [− 0.07, 1.02] vs − 0.04 [− 1.90, 0.11]). Median BP measurements across pregnancy are shown in Fig. 3. At Visit 3 (end of treatment), median BP appeared to be similar between L-citrulline-treated and placebo-treated groups; systolic BP 133 [*IQR* 124–144] vs 134 [130–140] mmHg; *p* = 0.7 and diastolic BP 83 [78–90] vs 90 [87–93]; *p* = 0.07.

There were no significant within group changes in any secondary cardiovascular outcomes calculated as change from baseline to end of treatment (data not shown). Pre-specified exploratory analyses investigating between group differences did not identify any significant differences (Online Resource 7). Median uterine artery pulsatility index and PWV across gestation are shown in Online Resource 8. The change in PWV was not different between groups (− 0.36 [− 0.9, 0.18] (*n* = 14) vs − 0.04 [− 1.22, 1.3]; *n* = 8).

### Exploratory Analysis

**Fig. 4:**
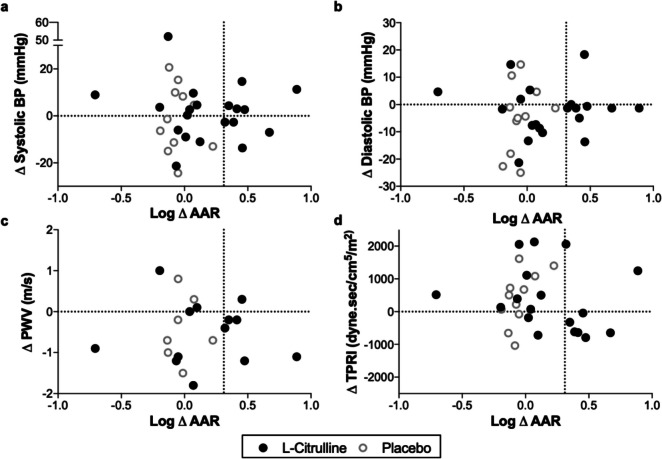
Change in peripheral cardiovascular parameters from baseline to the end of treatment by biochemical change in arginine:ADMA ratio (AAR). **a** Systolic BP. **b** Diastolic BP. **c** Pulse wave velocity. **d** Peripheral vascular resistance shown as total peripheral vascular resistance indexed for body surface area (TPRI). Change in cardiovascular parameter following 8 weeks of treatment is shown on the *y* axes; (log) delta AAR is shown on the *x* axes. The dashed vertical line indicates a significant increased (log) delta AAR ratio from baseline

To investigate the efficacy of L-citrulline supplementation on maternal cardiovascular function, the association between log change in AAR was plotted against the change in cardiovascular parameter between baseline and 8 weeks. There was no consistent reduction in BP (clinic or ambulatory), PWV or peripheral vascular resistance in participants in whom there was an increase in AAR (Fig. 4 and Online Resource 9). This finding was unchanged by the substitution of absolute AAR levels.

## Discussion

To our knowledge, this is the first randomised controlled trial (RCT) to investigate the effects of L-citrulline supplementation in a population of pregnant women with chronic hypertension. In this feasibility study, we have confirmed acceptability of the intervention and the study protocol, but we also highlighted significant issues with compliance and the timing of study visits which have complicated the interpretation of the clinical findings. We did not demonstrate a difference in diastolic BP following 8 weeks’ treatment (primary clinical outcome), nor a consistent effect of L-citrulline supplementation on maternal cardiovascular function.

Recruitment rate was high within our single centre (5.1 women/month) and the majority of women reported that “taking the allocated treatment” was “easy”. These data confirm the acceptability of the intervention to this group of women and support the feasibility of recruiting to a larger study. However, despite high acceptability, only 65.6% self-reported good compliance with the intervention (missing 1–2 doses/month or fewer), and this correlated well with biochemical measurements. Whilst qualitative measures of compliance were planned, these could not be completed due to inadequate return of treatment/placebo bottles. Further studies of this L-citrulline intervention must (i) seek to improve compliance through participant education or use of adherence aids, (ii) attempt to enforce more robust assessments of compliance and (iii) anticipate reduced compliance when calculating sample size.

Supplementation with 3g of L-citrulline twice daily was associated with a significant increase in plasma arginine, particularly in women who reported good compliance with the intervention. Accordingly, the AAR was increased at 4 weeks. This relationship did not persist at the final study visit, perhaps because the timing of the third visit in relation to completion of the intervention was inconsistent and some women had completed their treatment several days prior to their final visit. Alternatively, it is possible that compliance reduced over time. In studies outside of pregnancy, supplementation with the same dose of L-citrulline increased the plasma AAR and also resulted in elevated urinary nitrate and increased cGMP excretion [22], although these outcomes were not assessed here. Meta-analysis of trials in non-pregnant, mainly hypertensive subjects have demonstrated consistent reductions in systolic BP after 1–17 weeks of treatment with L-citrulline dose 1–9 g/day and in diastolic BP following treatment with ≥ 6 g/day (− 2.75 mmHg; *95% CI* [− 5.37, − 0.12]; *p* = 0.04) [27]. However, in the current RCT, we did not observe a significant reduction in systolic or diastolic BP after adjustment for baseline BP measurements. In a pre-specified exploratory analysis, increased AAR was not associated with improvement in a range of cardiovascular parameters. Whilst it should be acknowledged that the study was not powered for these parameters, taken in conjunction with lack of effect on the primary clinical outcome, the absence of this relationship is not encouraging for a successful clinical outcome in a larger-scale RCT of this intervention.

There could be several explanations for the negative effect on primary outcome reported in this study. Firstly, that there is no effect of L-citrulline treatment on BP in pregnant women with chronic hypertension, despite encouraging preliminary data [24, 25]. Secondly, that the study was underpowered to detect a small effect; this was predominantly a feasibility study to assess process outcomes, but was prospectively powered to detect a 6 (SD 10) mmHg within group change in diastolic BP with 36 participants. Problems with treatment availability, which curtailed recruitment, and discontinuation of treatment by one participant in each arm resulted in the number of participants completing therapy falling below that pre-specified in power calculations. The 6 mmHg change in diastolic BP is a large clinical effect over a short time period. In addition, the power calculation assumed, from relevant local data, that without treatment, the change in diastolic BP from baseline to 22 weeks would be 0 (SD 10) mmHg. However, in this small cohort, there was a 5.0 (SD 12.1) mmHg change in diastolic BP between the baseline and 8-week visit in the placebo group, and the sample size was therefore insufficient to detect anything smaller than 11 mmHg within group difference in diastolic BP. Furthermore, despite randomisation, baseline BP was clinically significantly lower in the L-citrulline group compared with the placebo group, which may have made a type 2 error more likely; however the potential for baseline differences in this small sample size was anticipated and therefore changes in BP were adjusted for baseline BP. Thirdly, it is possible that despite choosing a dosing regimen which can reduce BP outside of pregnancy [27], the L-citrulline dose administered was subtherapeutic for the participant population. In this study, amongst women reporting good compliance, the increase in steady state AAR (baseline 132.7 ± 36.8 to 4 weeks 198.1 ± 57.9) was smaller than in a previous pharmacokinetics/pharmacodynamics study demonstrating a therapeutic effect of the same L-citrulline regime (baseline AAR 186 ± 8 increased to 278 ± 14 at 7 days) [22]. Pregnancy introduces additional complexity to the pharmacokinetics of using L-citrulline to increase plasma L-arginine, which may be important for treatment efficacy. The extensive maternal physiological adaptations of pregnancy may impact upon absorption and uptake of L-citrulline or the metabolism and urinary excretion of NO pathway constituents. Argininosuccinate synthetase and arginases, enzymes which are respectively involved in the conversion of L-citrulline to L-arginine and conversion of L-arginine to ornithine, are known to be active within the placenta [28]. However, the implications of placental metabolism for bioavailability of L-citrulline and L-arginine in the maternal compartment have not been determined. Whilst pharmacokinetic studies of L-citrulline have been performed in healthy male subjects [29], to our knowledge there have been no such studies undertaken in pregnant cohorts, a common occurrence for many potential interventions.

The failure to perform appropriate early-phase experimental studies in pregnant women is often driven by a failure to recognise the impact of pregnancy physiology in pharmacokinetics or a desire to reduce intervention studies in pregnant women. However, this compromises the quality and reliability of clinical trials, paradoxically exposing an additional number of women to ineffective therapies and delaying or preventing effective new therapies from reaching clinical use. The role of this study was to test the feasibility of recruitment and intervention in preparation for a definitive RCT. The findings of this study do not support the continuation of a large-scale RCT using this experimental protocol. Rather, a robust assessment of the pharmacokinetics of L-citrulline treatment in pregnant women is indicated prior to further investigation in randomised trials.

## Conclusion

This feasibility study is the first to test L-citrulline as a new therapeutic for pregnant women with chronic hypertension, a clinical group who continue to experience high incidence of poor pregnancy outcomes [3]. Despite strong evidence from outside pregnancy and preclinical pregnancy studies supporting the biological rationale for the study and a study design informed by data from a well-characterised cohort of women with the phenotype of interest, this feasibility study has identified several barriers to conducting a large-scale study of this intervention. Despite high acceptability of the study design and intervention, compliance with the treatment regime was poor which impaired confidence in the results. In women with good compliance with L-citrulline, AAR was increased but to a smaller degree than reported in similar studies outside of pregnancy. The unquantified effects of pregnancy on L-citrulline metabolism may necessitate the use of a higher dose to achieve a consistent effect size for which a clinical study can be adequately powered. In this study, no effect of the intervention on maternal BP was identified. To avoid the risk of a Type 2 error and a missed opportunity for a highly plausible intervention to meet an important clinical need, pregnancy-specific pharmacokinetic/pharmacodynamic studies are recommended.

### Supplementary Information

Below is the link to the electronic supplementary material.

Supplementary file1 (PDF 635 KB)

Supplementary file2 (DOC 218 KB)

## Data Availability

The data that support the findings of this study are available from the corresponding author upon reasonable request.

## Abbreviations

AAR: Arginine:asymmetric dimethylarginine ratio
ABPM: Ambulatory blood pressure monitoring
ADMA: Asymmetric dimethylarginine
BP: Blood pressure
CCB: Calcium channel blocker
eNOS: Endothelial nitric oxide synthase
HDP: Hypertensive disorders of pregnancy
MAViS: Manchester Antenatal Vascular Service
NICOM: Non-Invasive Cardiac Output Monitor
NO: Nitric oxide
PlGF: Placental growth factor
PWV: Pulse wave velocity
RCT: Randomised controlled trial
s-Flt1: Soluble fms-like tyrosine kinase 1
TPRI: Total peripheral vascular resistance index

## Declarations

### Animal Studies

No animal studies were carried out by the authors for this article.
