# Supplementary material for: CHronic hypERtension and L-citRulline studY (CHERRY): an Early-Phase Randomised Controlled Trial in Pregnancy
Source: Reprod Sci. 2023 Oct 3;31(2):560–8. doi: 10.1007/s43032-023-01335-4 (PMC10827856; doi:10.1007/s43032-023-01335-4)

CHronic hypERtension and L-citRulline studY (CHERRY): an early-phase randomised controlled trial in pregnancy

Reproductive Sciences

Laura ORMESHER MBChB(Hons) PhD[a,b]*, Stephanie A WORTON MBChB(Hons) MRes PhD[a,b*], Ashley BEST BSc MSc[c], Susanna R DODD BSc MSc PhD[c], Alice DEMPSEY MBChB PhD[a,b], Elizabeth C COTTRELL BSc(Hons), PhD[b], Heather GLOSSOP BSc(Hons)[a,b], Catherine CHMIEL (BSc)[b], Hoi Yee WU BSc(Hons) MSc[a], Ben HARDWICK BSc MRes[c], Sophie HENNESSEY[c], Edward D JOHNSTONE MBChB PhD[a,b], Jenny E MYERS MB BS PhD[a,b].

a. Manchester University Hospitals NHS Foundation Trust, Manchester, UK.

b. Maternal and Fetal Health Research Centre, Division of Biology Medicine and Health, University of Manchester, Manchester, UK.

c. Liverpool Clinical Trials Centre, University of Liverpool, Liverpool, UK.

Corresponding author: Professor Jenny E Myers**.** Email: jenny.myers@manchester.ac.uk

Supplementary Tables and Figures (Online Resources 1-9)

Online Resource 1 Adverse events

**Allocation**	**Description**	**Severity**	**Relationship**	**SAE**	**Outcome**
L-Citrulline	Vomiting due to medication	Mild	Yes	No	Discontinued trial treatment. Consent gained for study follow-up.
Placebo	Discontinued trial treatment [*sic*] as feeling nauseous	Mild	Yes	No	Discontinued trial treatment. Consent gained for study follow-up.
Placebo	Congenital abnormality	Mild	No	Yes	Fetal ventriculomegaly diagnosed on fetal MRI at 33 weeks' gestation. Baby delivered at term for reasons not related to hypertension, well at birth, will have neonatal follow-up.
L-Citrulline	Nausea/vomiting began to increase after taking treatment	Mild	Yes	No	Missed 4 doses of treatment when could not tolerate.

Online Resource 2 Acceptability of treatment - questionnaire

Question	Answer	L-Citrulline n=22	Placebo n=10
**Q1: Was taking your allocated treatment?**	a) Easy	18 (81.8%)	8 (80.0%)
	b) Neither difficult or easy	2 (9.1%)	2 (20.0%)
	c) Difficult	2 (9.1%)	0 (0.0%)
**Q2: How would you describe the taste of the treatment you were given?**	a) Delicious	1 (4.5%)	0 (0.0%)
	b) Pleasant	12 (54.5%)	4 (40.0%)
	c) Unpleasant	8 (36.4%)	6 (60.0%)
	d) Awful	1 (4.5%)	0 (0.0%)
**Q3: How often did you miss a dose of your medication?**	a) Every day	2 (9.1%)	1 (10.0%)
	b) Once/twice per week	8 (36.4%)	2 (20.0%)
	c) Once/twice per month	4 (18.2%)	2 (20.0%)
	d) Hardly ever	10 (45.5%)	5 (50.0%)

Number (%). Questionnaires were missing for 4 participants.

Online Resource 3 Compliance with treatment – recall of missed doses

**Treatment**	**L-Citrulline**	**Placebo**
N	24	12
Number of missed doses	7 [2.5, 14.0]	4.5 [2.0, 27.0]
% missed doses	5.9 [2.4, 11.6]	3.1 [1.8, 23.3]

Median [Q1, Q3]

Online Resource 4 Citrulline, arginine, ADMA and arginine:ADMA ratio (AAR) at baseline through to the end of the study treatment. Biochemical measurements of citrulline (a), arginine (b) and ADMA (c) and the calculated arginine:ADMA ratio (AAR) (d) are shown prior to treatment (12-16 weeks; Visit 1), after 4 weeks' treatment (16-21 weeks; Visit 2) and at the completion of 8 weeks' treatment (21-24 weeks; Visit 3). Individual patient data is shown *(left)* with summary data shown as median and IQR *(right).* L-Citrulline and placebo-treated groups compared by mixed-effects analysis; *p<0.05 at Visit 2

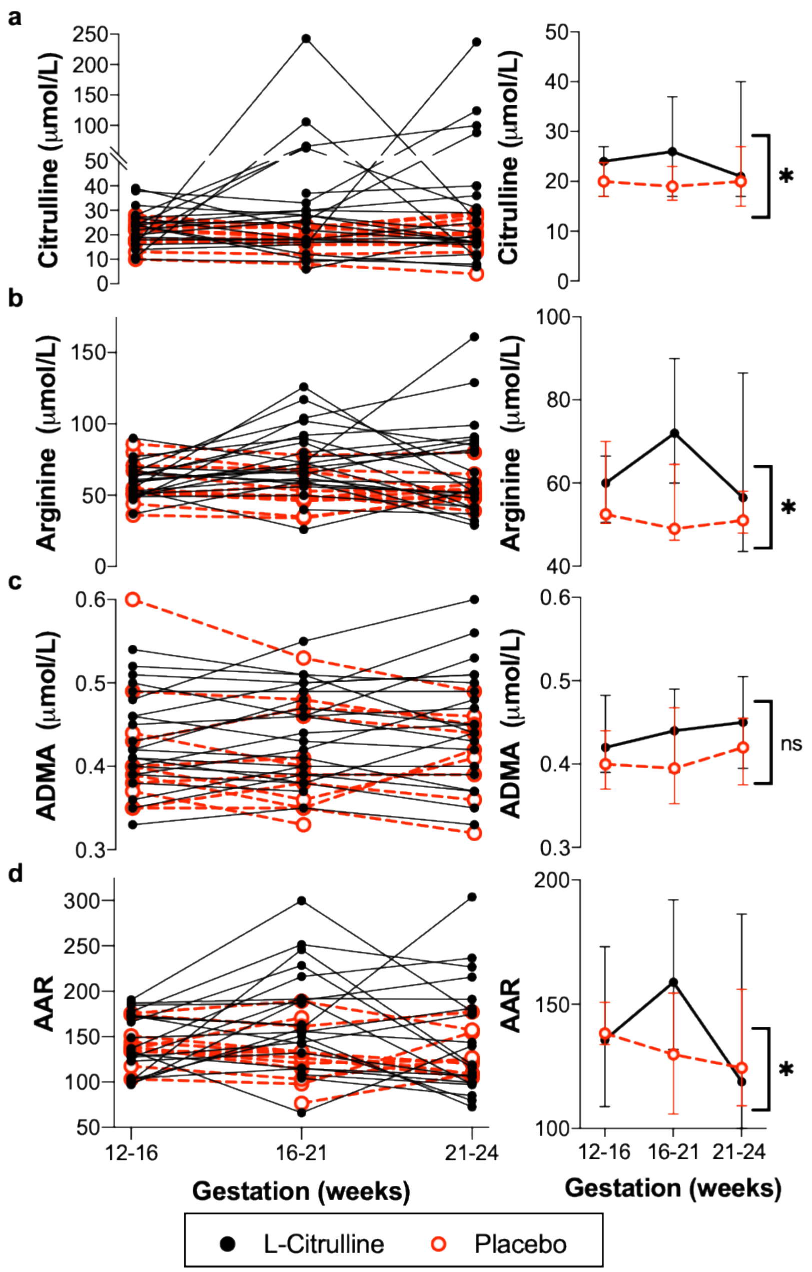

Online Resource 5 Pregnancy outcomes by treatment allocation

Pregnancy Outcome		L-Citrulline n=24	Placebo n=12
**Offspring Data**	Live born	24 (100%)	12 (100%)
	Female	14 (58.3%)	5 (41.7%)
	Male	10 (41.7%)	7 (58.3%)
	Gestational age (Days)	264.5 [236-285]	259.5 [239-281]
	Birthweight (grams)	2910 [1539-3910]	3405 [1912-4184]
**Delivery Medication**	Magnesium Sulphate required	1 (4.2%)	0
	Antenatal Steroids required	5 (20.8%)	2 (16.7%)
**Pregnancy Outcomes**	Pre-eclampsia	5 (20.8%)	3 (25.0%)
	Birthweight centiles	18.5 [0.18-90.6]	60.9 [3.2-99.6]
	SGA (<10th centile)	9 (40.9%)	2 (18.2%)
	FGR (<3rd centile)	5 (22.7%)	0 (0.0%)
	Gestational diabetes	4 (16.7%)	0

Number (%), median [min, max].

Online Resource 6 Changes to antihypertensive medication

		**L-Citrulline**<br>**n=24**	**Placebo**<br>**n=12**
**Medication decreased/stopped**	Visit 2	1 (4.2%)	0 (0.0%)
	Visit 3	0 (0.0%)	0 (0.0%)
**Medication increased**	Visit 2	1 (4.2%)	1 (8.3%)
	Visit 3	2 (8.3%)	1 (8.3%)

Number (%).

Online Resource 7 Secondary cardiovascular endpoints presented as change from baseline to end of treatment

Dependent Variable	Parameter	Estimate	Lower CL	Upper CL	N
**Visit 3 Systolic BP**	Intercept	73.15	53.53	92.78	
	Visit 1 Diastolic BP	0.55	0.39	0.58	
	Allocation L-citrulline	-0.28	-4.19	3.62	24
	Allocation placebo	0			12
	N Days*	0.03	-0.09	0.14	
**Visit 3 Diastolic BP**	Intercept	43.74	-5.4	92.89	
	Visit 1 Diastolic BP	0.41	0.04	0.78	
	Allocation L-citrulline	-0.64	-7.77	6.5	24
	Allocation placebo	0			12
	N Days*	0.1	-0.39	0.59	
**Visit 3 Central BP (SBPAO)**	Intercept	103.07	-4.97	211.11	
	Visit 1 Central BP	0.41	0.01	0.81	
	Allocation L-citrulline	1.69	-16.82	20.21	14
	Allocation placebo	0			8
	N Days*	-0.48	-2.06	1.1	
**Visit 3 Pulse Wave Velocity (PWV)**	Intercept	-4.4	-12.94	4.14	
	Visit 1 Pulse Wave velocity	1.18	0.61	1.75	
	Allocation L-citrulline	-0.42	-1.56	0.71	14
	Allocation placebo	0			8
	N Days*	0.05	-0.05	0.16	
**Visit 3 Normalised AIx**	Intercept	40.3	-91.35	171.96	
	Normalised AIO	0.95	-0.07	1.98	
	Allocation L-citrulline	-1.88	-27.46	23.7	14
	Allocation placebo	0			8
	N Days*	-0.69	-2.98	1.6	
**Visit 3 Cardiac Index**	Intercept	0.95	-0.88	2.79	
	Visit 1 Cardiac Index	0.17	-0.06	0.41	
	Allocation L-citrulline	0.03	-0.34	0.4	23
	Allocation placebo	0			12
	N Days*	0.02	-0.01	0.04	
**Visit 3 Stroke Volume Index**	Intercept	9.68	-13.02	32.37	
	Visit 1 Stroke Volume Index	0.14	-0.11	0.39	

Dependent Variable	Parameter	Estimate	Lower CL	Upper CL	N
	Allocation L-citrulline	1.93	-2.9	6.75	23
	Allocation placebo	0			12
	N Days*	0.18	-0.17	0.54	
**Visit 3 TRPI**	Intercept	3755.58	1047.6	6463.56	
	Visit 1 TRPI	0.2	-0.02	0.42	
	Allocation L-citrulline	-135.64	-738.18	466.89	23
	Allocation placebo	0			12
	N Days*	-11.56	-55.04	31.93	
**Visit 3 ABPM day systolic BP**	Intercept	8.13	-34.5	50.76	
	Visit 1 AMBP day systolic BP	1.06	0.84	1.28	
	Allocation L-citrulline	2.71	-3.2	8.63	20
	Allocation placebo	0			9
	N Days*	-0.31	-0.8	0.18	
**Visit 3 ABPM night systolic BP**	Intercept	32.19	-19.07	83.45	
	Visit 1 AMBP night systolic BP	0.98	0.74	1.21	
	Allocation L-citrulline	-3.33	-11.62	4.97	17
	Allocation placebo	0			7
	N Days*	-0.44	-1.08	0.2	
**Visit 3 ABPM day diastolic BP**	Intercept	26.55	-12.9	66	
	Visit 1 AMBP day diastolic BP	0.79	0.44	1.13	
	Allocation L-citrulline	-0.08	-6.04	5.88	20
	Allocation placebo	0			9
	N Days*	-0.19	-0.67	0.28	
**Visit 3 AMBP night diastolic BP**	Intercept	46.58	8.53	84.64	
	Visit 1 AMBP night diastolic BP	0.76	0.42	1.11	
	Allocation L-citrulline	-2.52	-8.78	3.74	17
	Allocation placebo	0			7
	N Days*	-0.46	-0.93	0.01	
**Visit 3 Uterine artery Resistance index**	Intercept	0.28	-0.06	0.63	
	Visit 1 artery resistance index	0.38	0.08	0.68	

Dependent Variable	Parameter	Estimate	Lower CL	Upper CL	N
	Allocation L-citrulline	0.01	-0.05	0.08	23
	Allocation placebo	0			12
	N Days*	0	0	0.01	
**Visit 3 Uterine artery Pulsatility index**	Intercept	0.33	-0.73	1.4	
	Visit 1 Pulsatility index	0.44	0.21	0.67	
	Allocation L-citrulline	-0.02	-0.24	0.21	23
	Allocation placebo	0			12
	N Days*	0	-0.02	0.02	

ANOVA including treatment allocation, days between visits and baseline measurement as covariates. ABPM: ambulatory BP monitoring. AIx: augmentation index corrected for heartrate. PWV: pulse wave velocity. TPRI: total peripheral vascular resistance index. SBPAO: central systolic blood pressure.

Online Resource 8 Uterine artery pulsatility index (a), pulse wave velocity (b) and angiogenic markers sFlt and PlGF (c) across pregnancy in women allocated to L-citrulline and placebo. Data shown as median [IQR]. Arrows indicate the start of treatment after the baseline visit and the end of treatment at/before Visit 3. Measurements taken beyond 24 weeks were recorded /obtained during routine clinical care visits

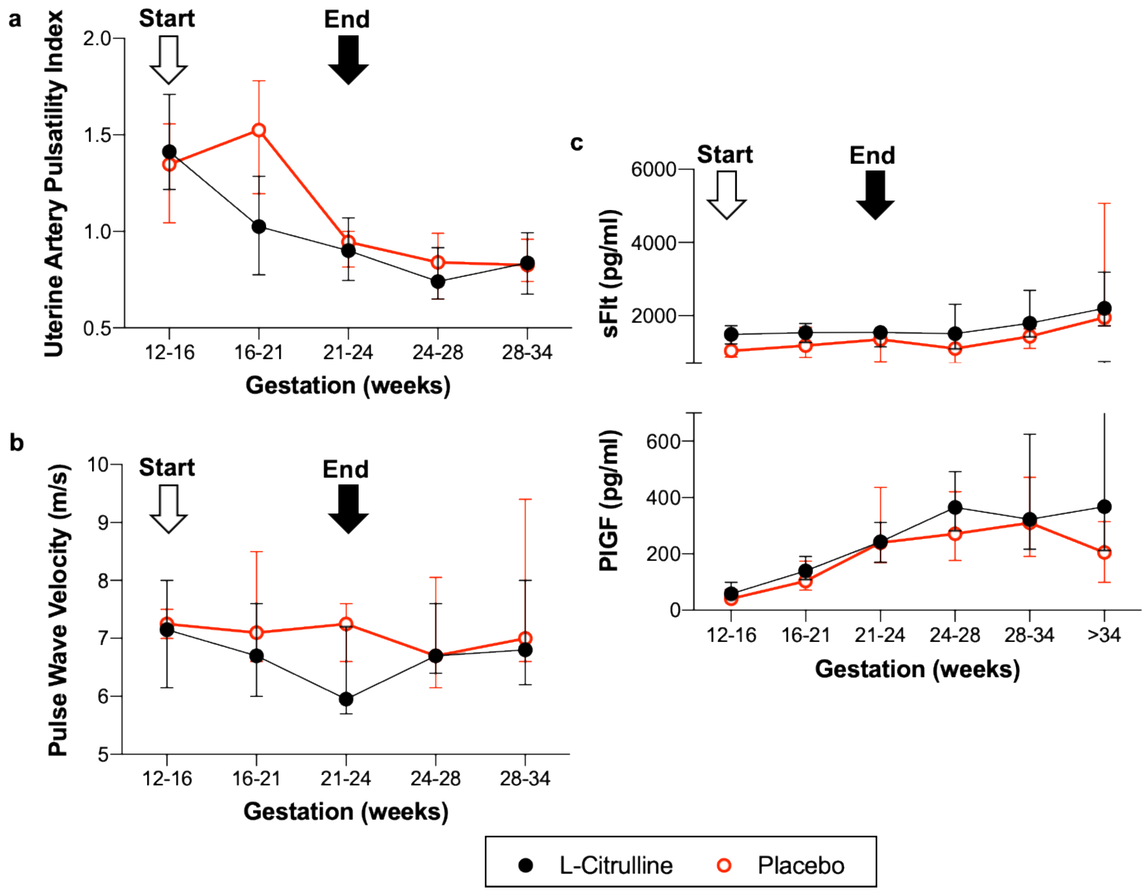

Online Resource 9 Change in ambulatory BP measurements from baseline to the end of treatment by biochemical delta in arginine:ADMA ratio (AAR): **(a)** Daytime systolic BP (sBP). **(b)** Daytime diastolic BP (dBP). **(c)** Nighttime sBP. **(d)** Nighttime dBP. Daytime average calculated from half hourly BPs between 08.00-22.00; Night time average calculated from hourly measurements between 22.00-08.00. The change in BP parameter following eight weeks of treatment is shown on the y axes; (log) delta AAR is shown on the x axes. The dashed vertical line indicates an increased (log) delta AAR from baseline

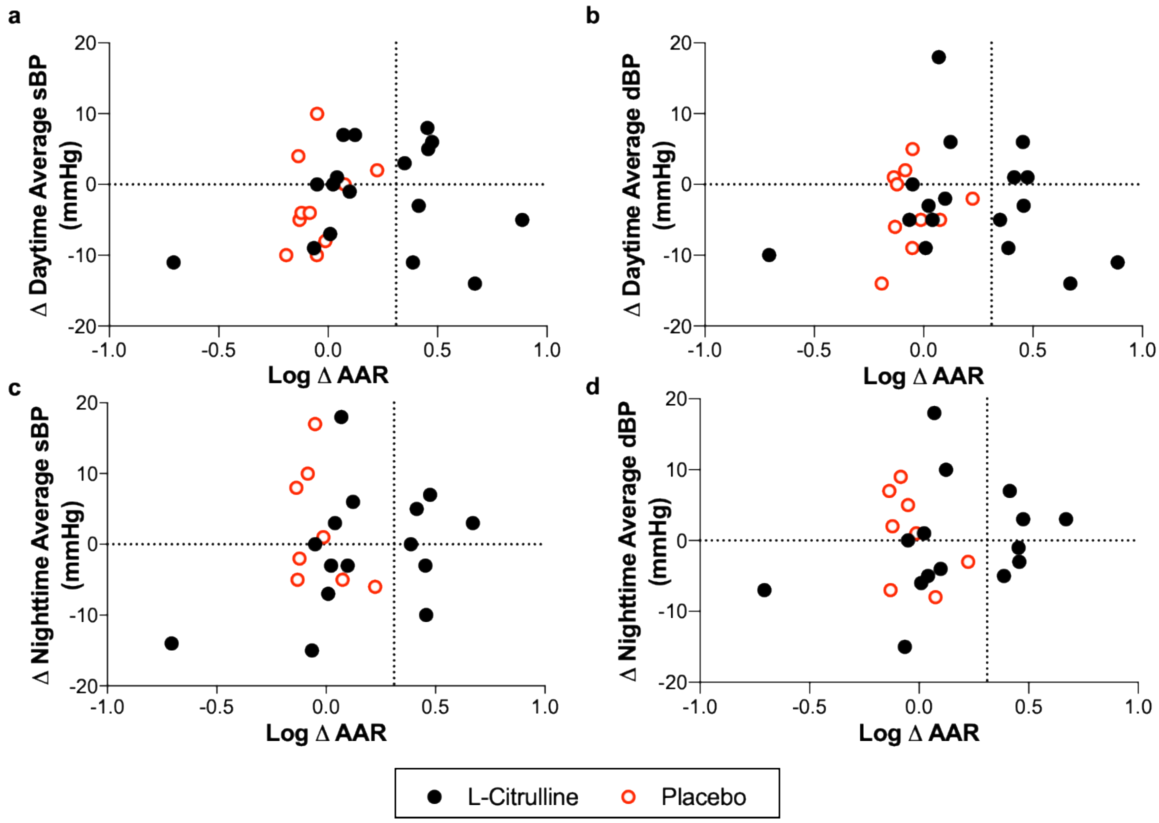

Supplement: Supplementary file 1 — Supplementary file1 (PDF 635 KB) [file 43032_2023_1335_MOESM1_ESM.pdf]
